# Gait speed as a predictor of mortality in older men with cancer: A longitudinal study in Peru

**DOI:** 10.1016/j.heliyon.2022.e08862

**Published:** 2022-01-29

**Authors:** Elizabeth Dociak-Salazar, José L. Barrueto-Deza, Diego Urrunaga-Pastor, Fernando M. Runzer-Colmenares, José F. Parodi

**Affiliations:** a Universidad Científica del Sur, Facultad de Ciencias de la Salud, Carrera de Medicina Humana, Lima, Peru; b Universidad de San Martin de Porres, Facultad de Medicina Humana, Centro de Investigación del Envejecimiento (CIEN), Lima, Peru; c Bamboo Senior Health Services, Lima, Peru

**Keywords:** Gait speed, Frailty, Cancer, Mortality, Older adults, Latin America

## Abstract

**Background:**

Given the increase in incidence and mortality from cancer in recent years in Latin America and Peru, it is necessary to identify frailty older adults at higher risk of disability, hospitalizations and mortality. However, its measure is complex and requires time. For this reason, it has been proposed that frailty can be evaluated by a single measure, as gait speed. We aimed to evaluate the role of gait speed as a predictor of mortality in older men with cancer in Peru.

**Methods:**

A prospective cohort study was carried out that included military veterans (aged 60 years and older) with an oncological diagnosis evaluated at the *Centro Médico Naval* in Peru during the period 2013–2015. Slow gait speed was defined as <0.8 m/s. All-cause mortality was recorded during a 2-year follow-up. Sociodemographic characteristics, medical and personal history, and functional assessment measures were collected. We performed Cox regression analysis to calculate hazard ratios with their respective 95% confidence intervals.

**Results:**

922 older men were analyzed from 2013 to 2015, 56.9% (n = 525) of whom were >70 years of age. 41.3% (n = 381) had slow gait speed with a mortality incidence of 22.9% (n = 211) at the end of follow-up. The most frequent types of cancer in the participants who died were of the lung and airways (26.1%), liver and bile ducts (23.2%), and lymphomas and leukemias (16.6%). In the adjusted Cox regression analysis, we found that slow gait speed was a risk factor for mortality in older men with cancer (adjusted hazard ratio = 1.55; 95% confidence interval: 1.21–2.23).

**Conclusions:**

Slow gait speed was associated with an increased risk of mortality in older men with cancer. Gait speed could represent a simple, useful, inexpensive, rapidly applicable marker of frailty for the identification of older men at higher risk of mortality. Gait speed could be useful in low- and middle-income countries, and in rural areas with limited access to health services.

## 1 Introduction

Cancer is a global problem, and it is estimated that in 2018 there were approximately 18.1 million new cases and a total of 9.6 million deaths from this disease [1]. Likewise, the number of new cases is expected to increase to 24 million by 2035 [2]. Among the main causes of this situation are modifiable factors such as unhealthy and non-modifiable lifestyles, such as age. It should be noted that it is estimated that the number of older adults will double in the next two decades worldwide [3], and considering that the incidence of cancer increases with age, an increase in the number of older adults diagnosed with cancer worldwide is expected in the coming years.

With the increase in incidence and mortality by cancer in older adults, identification of groups at higher risk is challenging. Therefore, evaluation of elderly subjects with cancer is recommended using Comprehensive Geriatric Assessment (CGA), a multicomponent tool that includes the evaluation of functionality, nutritional status, social support, cognitive function, polypharmacy and frailty in the elderly [4]. However, it could not be applicable in external consultation, where we have limited time. Frailty is relevant and is defined as an excessive vulnerability to stressors and a lower capacity to maintain or recover homeostasis in the event of an adverse event [5]. Despite having an accepted definition for this concept, there is no consensus regarding its evaluation [6]. Frailty allows identifying groups at higher risk of disability, hospitalizations and mortality, as well as adverse effects due to treatment and postoperative complications in cancer patients [7]. However, one of the most accepted methods for measuring frailty is the Fried frailty phenotype, the definition of which includes a series of criteria [8], which require more time, complexity and cost to apply. For this reason, it has been proposed that frailty can be evaluated by a single measure, as gait speed, which have a high diagnostic value according to previous studies [9, 10].

The role of slow gait speed as a predictor of mortality in older adults with cancer has been evaluated in previous studies in Europe and the United States [11, 12, 13, 14]. One systematic review evaluated its usefulness as a predictor of adverse outcomes; however, the results were not conclusive in older adults with cancer [6]. Given the increase in incidence and mortality from cancer in recent years in Latin America [15] and Peru [16], screening tools for frailty that are easy to apply, reproducible and inexpensive are needed. Gait speed has been described as a marker of frailty [10] and its implementation could be very useful in low- and middle-income countries, and especially in rural areas with limited access to health services.

Military veterans are an interesting population, they were exposed to great physical training and muscle stress during their lives [17], and they usually have a high prevalence of chronic physical and mental illnesses [18, 19, 20]. Then, previous studies have described the high prevalence of frailty and impaired gait speed as predictors of adverse outcomes in military veterans [21, 22, 23]. Nonetheless, to date no study has evaluated slow gait speed as a predictor of mortality in older adults with cancer in Latin America. Therefore, the present study aimed to evaluate the role of slow gait speed as a predictor of mortality in older men with cancer in Peru.

## 2 Material and methods

### 2.1 Study design, population and sample

We performed a secondary data analysis from a prospective cohort study that included military veterans (men aged 60 years and older) with an oncological diagnosis and curative therapeutic indications, evaluated and enrolled in 2013 and followed until 2015, in the *Geriatrics service of Centro Médico Naval (CEMENA) “Cirujano Mayor Santiago Tavara*”, in Callao, Peru. Previous studies have been carried out with this database [24, 25, 26]. In the initial evaluation, we had 1178 potentially eligible participants, however, we excluded 81 due to dementia, 121 for having a score ≤23 in the Mini Mental State Examination, 4 due to incomplete data in the medical records, 9 because they will receive treatment without curative intention, 4 due to treatment discontinuation, 6 were lost during follow-up and 31 did not agree to participate in the study. We also excluded participants who did not have the variables of interest. We calculated a power of 100% for this secondary data analysis considering a hazard ratio (HR) of 2.0 based on the results by Nofuji *et al* [27] in a sample of 922 participants.

### 2.2 Procedures

The patients were enrolled after confirming the oncological diagnosis. In the first geriatric consultation, sociodemographic variables, medical history and performance-based measures, including gait speed were collected. Likewise, all-cause mortality was considered along the follow-up period. The variables of interest were measured by the application of scales and performance-based measures.

### 2.3 Variables

#### 2.3.1 Outcome variable

All cause-mortality was evaluated according to the registry of the epidemiological surveillance office of CEMENA during the follow-up period.

#### 2.3.2 Exposure variable

Gait speed was determined as the time necessary to reach a distance of four meters. The four meters distance was divided by time to obtain the gait speed per meter. Slow gait speed was defined as a speed less than 0.8 m/s or as individuals who could not complete the test [28]. The highest gait speed time of each participant was considered.

#### 2.3.3 Other variables

##### 2.3.3.1 Sociodemographic characteristics

Age (60–70, ≥71 years) and marital status (single, married/cohabiting, divorced/separated, widowed) were considered. These variables were collected through self-reporting.

##### 2.3.3.2 Medical and personal history

Comorbidities included hypertension, type 2 diabetes mellitus, chronic kidney disease, chronic obstructive pulmonary disease, overweight/obesity (defined by a body mass index ≥25 kg/m^2^ and ≥30 kg/m^2^, respectively), osteoporosis and dyslipidemia. Urinary incontinence (yes/no) was assessed using the Edmonton frailty scale [29], as well as sedentary lifestyle (yes/no), defined as a score less than 64 on the Physical Activity Scale for the Elderly (PASE) [30, 31]. These variables were collected from the medical records. A variable was generated that consolidated the previously described diseases and conditions of the elderly (0, 1, ≥2).

Polypharmacy was defined as the consumption of five or more drugs under medical prescription [32], and the use of health services was defined as admission to the CEMENA hospitalization or emergency service (yes, no).

Smoking history (yes, no), family history of cancer (yes/no) and falls in the last year (yes, no) were considered as part of the personal history. This information was collected through self-reporting.

##### 2.3.3.3 Functional assessment

The Barthel index was used to evaluate dependency activities of daily living (ADL). This index evaluates 10 activities, with a score between 0 and 100 [33]. Participants with a score less than 100 were considered dependent. The nutritional status of the study participants was included using the Mini Nutritional Assessment scale, defining malnutrition as a score less than 17 [34]. Dynapenia was defined as grip strength less than 26 kg [28].

We evaluated exhaustion with three questions that evaluated how the older adult felt during the last two weeks: a) Do you feel full of energy? (yes/no); b) Do you feel like you cannot go on? (yes/no); c) Do you feel that everything you do is an effort? (yes/no). A score greater than or equal to 2 was considered positive [8].

We included self-reported weight loss, which was assessed using the Edmonton frailty scale (yes/no) [29].

### 2.4 Statistical analysis

We analyzed the data using the statistical package STATA v14.0. Descriptive results are presented using absolute and relative frequencies. In addition, we described quantitative variables using means ± standard deviation (SD). The bivariate analysis was performed using the Chi-square and Fisher exact tests to analyze categorical covariates and outcomes. A crude and adjusted Cox regression analysis was performed (after evaluated the model's assumptions as constant and proportional hazards) to evaluate the association between gait speed as a predictor of mortality in the study sample. The adjusted model included the covariates with a p < 0.05 in the crude Cox regression model. In addition, we performed a sensitivity analysis including age as a risk factor for all-cause mortality. Crude and adjusted HRs with their respective 95% confidence intervals (95% CI) were estimated. We elaborated a Kaplan-Meier curve to graph the participants' survival according to gait speed groups, and we compared them using the Log-rank test.

### 2.5 Ethical aspects

This study was evaluated and approved by the Institutional Review Board of the *Universidad Científica del Sur* (409-2020-PRE15). Likewise, the primary study was approved by the CEMENA Ethics Committee (Memorandum N°CEI-CMN-134-2009). The participants signed informed consent prior to entering the study.

## 3 Results

### 3.1 Characteristics of the study sample and bivariate analysis

A total of 922 older men with cancer were analyzed, with a mean follow-up of 589 days, all of whom were male, and 56.9% (n = 525) were older than 70 years, while 55.6% (n = 513) were married or cohabiting. Likewise, 92.6% (n = 854) had at least one comorbidity, and only 24.2% (n = 223) were malnourished. On the other hand, 55.4% (n = 511) had polypharmacy, 46.8% (n = 431) reported having perceived weight loss, and 48.7% (n = 449) had dynapenia. We found a gait speed mean of 0.90 ± 0.10 (SD) in the study sample. In addition, we found a mean of 0.90 ± 0.11 (SD) and 0.75 ± 0.11 (SD) in the normal and slow gait speed group, respectively. Slow gait speed was presented by 41.3% (n = 381), and the incidence of mortality was 22.9% (n = 211). The median follow-up of the normal and slow gait speed group was 667.8 (IQR: 34.0) and 379.1 (IQR: 22.1) days, respectively (Table 1).

**Table 1:** Descriptive and bivariate analysis of the study sample according to all-cause mortality (n = 922).

Variables	n	%	Mortality after 2 years of follow-up		P-value
			No 77.1% (n = 711)	Yes 22.9% (n = 211)	
Age					0.06
60–70 years	397	43.1	318 (44.7)	79 (37.4)	
≥71 years	525	56.9	393 (55.3)	132 (62.6)	
Marital status					<0.001
Single	179	19.4	161 (22.6)	18 (8.5)	
Married/cohabiting	513	55.6	484 (68.1)	29 (13.7)	
Divorced/separated	109	11.8	26 (3.7)	83 (39.3)	
Widowed	121	13.1	40 (5.6)	81 (38.4)	
Comorbidities					<0.001
0	68	7.4	26 (3.7)	42 (19.9)	
1	308	33.4	244 (34.3)	64 (30.3)	
≥2	546	59.2	441 (62.0)	105 (49.8)	
Smoking habit					<0.001
No	738	80.04	650 (91.4)	88 (41.7)	
Yes	184	19.96	61 (8.6)	123 (58.3)	
Malnutrition					<0.001
No	699	75.8	600 (84.4)	99 (46.9)	
Yes	223	24.2	111 (15.6)	112 (53.1)	
Functional dependence for ADL^1^					<0.001
No	673	73.0	603 (84.8)	70 (33.2)	
Yes	249	27.0	108 (15.2)	141 (66.8)	
Polypharmacy					<0.001
No	411	44.6	345 (48.5)	66 (31.3)	
Yes	511	55.4	366 (51.5)	145 (68.7)	
Use of health services					<0.001
No	477	51.7	405 (57.0)	72 (34.1)	
Yes	445	48.3	306 (43.0)	139 (65.9)	
Self-reported weight loss					<0.001
No	491	53.2	389 (54.7)	102 (48.3)	
Yes	431	46.8	322 (45.3)	109 (51.7)	
Exhaustion					<0.001
No	543	58.9	449 (63.2)	94 (44.5)	
Yes	379	41.1	262 (36.8)	117 (55.5)	
Dynapenia					<0.001
No	473	51.3	403 (56.7)	70 (33.2)	
Yes	449	48.7	308 (43.3)	141 (66.8)	
Falls in the last year					<0.001
No	520	56.4	438 (61.6)	82 (38.9)	
Yes	402	43.6	273 (38.4)	129 (61.1)	
Gait speed					<0.001
Normal	541	58.7	471 (66.2)	70 (33.2)	
Slow	381	41.3	240 (33.8)	141 (66.8)	

1 Activities of daily living.

We found that slow gait speed was presented by 66.8% (n = 141) of the participants who died versus 33.8% (n = 240) of those who did not die. Likewise, statistically significant differences were found between study covariates and mortality in older men, with the exception of marital status (Table 1).

### 3.2 Mortality according to type of cancer

The most frequent types of cancer in the study sample were prostate (20.5%), colorectal (17.7%), stomach (15.7%), skin (10.7%) and lung and airways (9.8%). However, the most frequent types of cancer in the participants who had slow gait speed were colorectal (24.0%), stomach (22.2%), lungs and airways (13.5%), liver and bile ducts (12.1%) and multiple myeloma (9.2%). In addition, the most common types of cancer in the group who died were lung and airways (26.1%), liver and bile ducts (23.2%), lymphomas and leukemias (16.6%), multiple myeloma (16.1%) and colorectal (9.0%) (Table 2).

**Table 2:** Frequency of mortality according to type of cancer.

Type of cancer	n	%	Slow gait speed (n = 379)		Mortality (n = 211)	
			n	%	n	%
Lungs and airways	90	9.8	51	13.5	55	26.1
Liver and bile ducts	88	9.5	46	12.1	49	23.2
Lymphomas and leukemias	54	5.9	27	7.1	35	16.6
Multiple myeloma	58	6.3	35	9.2	34	16.1
Colorectal	163	17.7	91	24.0	19	9.0
Stomach	145	15.7	84	22.2	18	8.5
Prostate	189	20.5	31	8.2	1	0.5
Skin	99	10.7	11	2.9	0	0
Urogenital/Others	36	3.9	3	0.8	0	0

### 3.3 Gait speed as a predictor of mortality

**Figure 1:**
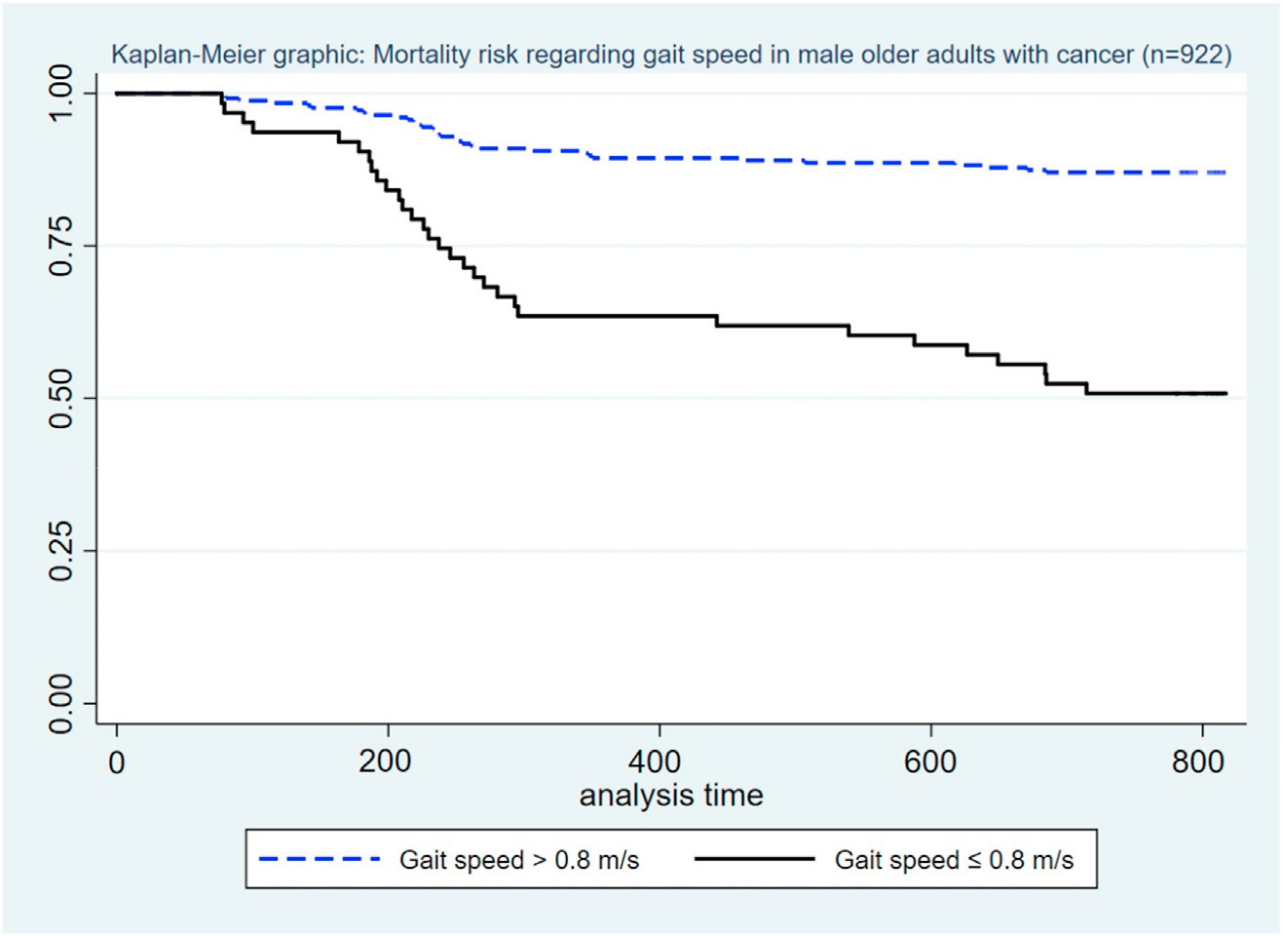
Kaplan-Meier survival curves according to gait speed groups in male older adults with cancer.

In the crude Cox regression analysis, we found that slow gait speed was a risk factor for mortality in older men with cancer (cHR = 2.13; 95%CI: 1.34–3.49). This association remained statistically significant in the adjusted Cox regression analysis independently of confounders (aHR = 1.55; 95%CI: 1.21–2.23) (Table 3). Likewise, the confounders that remained statistically significantly associated in the adjusted model to assess risk factors for mortality in older men with cancer were marital status, smoking history, functional dependence for ADL, polypharmacy, dynapenia and falls in the last year. In addition, we performed an additional adjusted model as a sensitivity analysis including age despite not having a statistically significant association in the crude model (Table 4). We found a higher mortality risk in the depressed frail phenotype group according to the Kaplan-Meier curve (p < 0.001) (Figure 1).

**Table 3:** Cox regression to assess risk factors for all-cause mortality in the study sample (n = 922).

Variables	Crude model	Adjusted model
	cHR^1^ (95%CI)	aHR^1^ (95%CI)
Gait speed		
Normal	1	1
Slow	**2.13 (1.34–3.49)**	**1.55 (1.21–2.23)**
Age		
60–70 years	1	
≥71 years	1.26 (0.88–1.43)	Not included
Marital status		
Single	1	1
Married/cohabiting	**0.60 (0.55–0.76)**	**0.69 (0.58–0.79)**
Divorced/separated	**1.34 (1.22–1.48)**	1.12 (0.92–1.49)
Widowed	**4.33 (2.39–6.01)**	**1.19 (1.09–1.71)**
Comorbidities		
0	1	
1	0.89 (0.80–1.39)	
≥2	0.96 (0.83–1.41)	Not included
Smoking habit		
No	1	1
Yes	**2.11 (1.09–2.35)**	**1.13 (1.03–1.38)**
Malnutrition		
No	1	
Yes	0.88 (0.86–1.29)	Not included
Functional dependence for ADL^2^		
No	1	1
Yes	**4.17 (2.67–5.09)**	**1.71 (1.36–2.82)**
Polypharmacy		
No	1	1
Yes	**1.70 (1.36–2.00)**	**1.21 (1.16–1.40)**
Use of health services		
No	1	1
Yes	**2.06 (1.99–2.32)**	1.13 (0.99–1.36)
Self-reported weight loss		
No	1	
Yes	1.21 (0.97–1.47)	Not included
Exhaustion		
No	1	1
Yes	**1.78 (1.71–2.36)**	1.05 (0.72–2.12)
Dynapenia		
No	1	1
Yes	**2.15 (1.54–2.34)**	**1.39 (1.15–1.43)**
Falls in the last year		
No	1	1
Yes	**2.32 (1.23–3.45)**	**1.44 (1.21–3.21)**

^1^ ​HR: Hazard Ratio; ^2^ ADL: Activities of daily living. Statistically significant results are in bold.

**Table 4:** Cox regression sensitivity analysis including age to assess risk factors for all-cause mortality in the study sample (n = 922).

Variables	Adjusted model
	aHR^1^ (95%CI)
Gait speed	
Normal	1
Slow	**1.51 (1.20–1.98)**
Age	
60–70 years	1
≥71 years	1.21 (0.71–1.23)
Marital status	
Single	1
Married/cohabiting	0.63 (0.51–1.02)
Divorced/separated	1.12 (0.91–1.45)
Widowed	1.05 (0.94–1.41)
Comorbidities	
0	
1	
≥2	Not included
Smoking habit	
No	1
Yes	**1.04 (1.01–1.13)**
Malnutrition	
No	
Yes	Not included
Functional dependence for ADL^2^	
No	1
Yes	**1.61 (1.31–1.92)**
Polypharmacy	
No	1
Yes	**1.13 (1.06–1.34)**
Use of health services	
No	1
Yes	1.14 (0.96–1.22)
Self-reported weight loss	
No	
Yes	Not included
Exhaustion	
No	1
Yes	1.01 (0.89–1.44)
Dynapenia	
No	1
Yes	**1.21 (1.05–1.27)**
Falls in the last year	
No	1
Yes	**1.37 (1.20–2.90)**

^1^ ​HR: Hazard Ratio; ^2^ ADL: Activities of daily living. Statistically significant results are in bold.

## 4 Discussion

### 4.1 Main results

The results of this study show that slow gait speed in older men with cancer increases the risk of mortality by approximately 1.5-fold. The types of cancer with the highest incidence of mortality were lung and airways, liver and bile ducts, lymphomas and leukemias, and multiple myeloma. The risk factors for mortality in this population were marital status, polypharmacy, functional dependence for ADL, dynapenia, smoking habit and falls in the last year.

### 4.2 Comparison with literature

In this study, slow gait speed was found to be a risk factor for mortality in older men with cancer in Peru. This finding agrees with previous studies carried out in older adults in France, which included older adults with local, locally-advanced and metastatic cancer, however, the follow-up was shorter, and the sample size was also smaller [13, 35]. On the other hand, while some studies have been performed in older adults in the United States, the evaluation of gait speed was not made using a walking time to go four meters [36]. Similar to our results, a previous multinational study performed a pooled analysis of nine cohorts, finding that gait speed was a risk factor for mortality, independently of confounders [37]. Likewise, other studies have evaluated the survival of older adults with cancer who had slow gait speed, but only included patients with brain and hematological cancer, respectively [11, 38]. This is important to take into account since while the incidence of these types of cancer is not high, the mortality is [2], and thus, these studies reported a greater magnitude of association. Our study, however, was carried out in a male military veterans population, who could present better physical performance due to their military training [39]. In the previously mentioned studies, excepting the multinational study, the sample size and follow-up time were shorter, and they did not include relevant confounders such as falls, nutritional status and disability in the adjusted regression model, as in this study. This variables have been described as relevant predictors of mortality in older adults with cancer [40, 41].

To our knowledge this is the first study to evaluate the association between slow gait speed and mortality in older men with cancer in Latin America. This is worrying because Latin America is a region with a high mortality rate from cancer [15]. This situation could be explained by the aging of the population, the increase in comorbidities, sedentary lifestyle and unhealthy habits that are increasing in the region [42]. In addition, it is of note that in Peru one in every five deaths can be attributed to cancer, and the mortality rate is higher in older adults [16]. Likewise, in older men, the type of cancer with the highest mortality described in the literature is that of the lungs and stomach [16], which is consistent with what was found in the present study. Other studies have highlighted mortality caused by hematological cancer [11], which was also associated with a high incidence of mortality in our study.

The current study found that slow gait speed increased the risk of mortality in older men with cancer, which could be due to multiple explanations. Five out of ten older men in this study had polypharmacy, which has been described as a risk factor for mortality and was associated in the adjusted analysis. This situation could be explained in that comorbidities decrease the functional and cognitive reserve, increasing the risk of mortality, especially in older adults with cancer [43]. Likewise, polypharmacy can increase the risk of adverse effects, frailty, hospitalizations and mortality. On the other hand, the presence of comorbidities has been associated with a higher incidence of cancer, which can increase mortality in older adults [44]. Finally, one of the main manifestations of a decrease in cognitive reserve and a marker of frailty is an alteration in gait speed, which can be more affected during an oncological process [41].

In addition, six out of ten older men who died were dependent on ADL, and this was a risk factor for mortality in the study sample, in agreement with what has been described in previous studies [13, 36]. Disability related to ADL is part of the CGA and assesses domains other than those that measure frailty, in addition to being a predictor of chemotherapy toxicity making evaluation of this factor important [7]. Likewise, gait speed and other frailty markers such as poor physical performance and altered grip strength have been associated with a higher incidence of ADL disability [45, 46], making preventive measures important due to the risk of fatal outcomes.

A history of falls in the last year and dynapenia were found to be risk factors for mortality in older men, which is consistent with previous studies and can be explained in a similar manner. Falls are nonspecific manifestations of the presence of a disease or alteration of homeostasis in the elderly, as in the case of an oncological disease [45, 47]. Antineoplastic treatment may affect older adults with a low functional reserve and dynapenia to a greater extent [45, 47]. These conditions are preventable and the risk of mortality can be reduced in this population group [40].

Divorced, separated or widowed participants were found to have twice the risk of mortality than married individuals. Previous studies have described that married couples have a lower mortality [48, 49], which could be due to the greater family support and less social isolation [47, 50]. These two components have been described as risk factors for the incidence of cancer, poor tolerance to treatment, and mortality from this disease [51].

In our study, we found that the smoking habit increased the risk of mortality, which is consistent with previous studies. Smoking has been described as playing a relevant role in the pathophysiology of frailty, probably due to its association with cardiovascular, respiratory and cancer comorbidities, which can lead to a greater risk of disability and, finally, frailty [52]. On the other hand, cigarette smoke contains a high number of toxic chemicals which generate an increase in inflammation markers, which with chronic use can lead to loss of muscle mass, exhaustion, weakness, slower gait speed and greater frailty [53]. Smoking has also been associated with a higher incidence of cancer, with special involvement of the lung, upper respiratory tract and upper digestive tract [54, 55], which were the types of cancer with the highest mortality in this study.

### 4.3 Clinical perspectives

Gait speed is a simple and fast measure that can be applied at the hospital level, as well as at the first level of medical care, in addition to being an adequate, low-cost and highly practical alternative in the geriatric population with cancer [6]. Previous systematic reviews have evaluated the role of gait speed as a predictor of disability, falls, hospitalization and mortality in older adults; the results, however, were not conclusive in the oncological population, suggesting the need for longitudinal studies to evaluate its usefulness as a marker of frailty in this most vulnerable group [6]. Slow gait speed in older adults with cancer is related to the low functional reserve of these individuals leading to greater risk of adverse effects due to palliative therapy, as well as hospitalizations and mortality. Then, previous studies has described gait speed as a useful marker of frailty and it could be a functional tool for clinical practice and therapeutic decisions in older adults with cancer [10, 56].

### 4.4 Strength and weakness

This study has several limitations: 1) the follow-up time was 2 years, which could have limited the recording of a greater number of events; 2) Only male participants were included in the study sample, and the association may vary in female patients; 3) There is no record of the treatment doses received by the participants, which could affect the association of interest studied; 4) There were no relevant variables such as renal failure markers (urea and creatinine), which could serve to identify patients at higher risk of adverse effects to antineoplastic treatment and who could present a higher risk of mortality; 5) The study population was exclusively made up of retired marines, and therefore, the results cannot be extrapolated to the general population. Despite the limitations described, this study is one of the first carried out in Latin America that has evaluated the role of gait speed as a predictor of mortality in older men with cancer, who present a higher risk of fatal outcomes and prioritizes the identification of vulnerable groups. Likewise, the results of this study may have clinical utility given the need for an instrument that is easy to access, low in cost, and can be quickly applied in contrast to more complex frailty measurements. Prospective studies are needed to evaluate the association between gait speed and mortality in older adults of both sexes and include laboratory markers as well as antineoplastic treatment.

## 5 Conclusions

The study found that slow gait speed was associated with an increased risk of mortality in older men with cancer. In addition, it was observed that marital status, smoking history, functional dependence for ADL, polypharmacy, dynapenia and falls were risk factors for mortality. Gait speed could represent a useful, inexpensive, and quickly applied alternative for the identification of frail and vulnerable older men who could be at greater risk of mortality.

## Declarations

### Author contribution statement

Elizabeth Dociak-Salazar and José L. Barrueto-Deza: Conceived and designed the experiments; Wrote the paper.

Diego Urrunaga-Pastor: Conceived and designed the experiments; Analyzed and interpreted the data; Wrote the paper.

Fernando M. Runzer-Colmenares: Conceived and designed the experiments; Analyzed and interpreted the data; Contributed reagents, materials, analysis tools or data; Wrote the paper.

José F. Parodi: Analyzed and interpreted the data; Contributed reagents, materials, analysis tools or data; Wrote the paper.

### Funding statement

This research did not receive any specific grant from funding agencies in the public, commercial, or not-for-profit sectors.

### Data availability statement

The authors do not have permission to share data.

### Declaration of interests statement

The authors declare no conflict of interest.

### Additional information

No additional information is available for this paper.
